# Hybrid parastomal endoscopic repair (HyPER): a retrospective case series of 200 patients treated over ten years at a single center

**DOI:** 10.1007/s00464-025-12318-8

**Published:** 2025-11-25

**Authors:** Marek Szczepkowski, Mateusz Zamkowski, Bartosz Ziółkowski, Piotr Czyżewski, Piotr Witkowski, Maciej Śmietański

**Affiliations:** 1 Clinical Department of Colorectal, General and Oncological Surgery, Centre of Postgraduate Medical Education in Warsaw, Bielanski Hospital, Warsaw, Poland; 2 Department of General Surgery and Hernia Center, Swissmed Hospital, Wileńska 44, 80-215 Gdańsk, Poland; 3 II Department of Radiology, Medical University of Gdańsk, Gdańsk, Poland; 4 Faculty of Rehabilitation, Józef Piłsudski University of Physical Education in Warsaw, Warsaw, Poland; 5 Śmietański Hernia Center, LUX MED Hospital, Wileńska 44, 80-215 Gdańsk, Poland

**Keywords:** Parastomal hernia, Abdominal wall reconstruction, Hybrid parastomal endoscopic repair, HyPER, Case series, Hernia surgery

## Abstract

**Introduction:**

Parastomal hernia is a common and challenging complication after stoma formation, often requiring complex surgical management. To address limitations of conventional techniques, we developed the Hybrid Parastomal Endoscopic Repair (HyPER) technique, which combines laparoscopic and open approaches. This case series aimed to evaluate the long-term safety, efficacy, and technical considerations of the HyPER method in a large, consecutive cohort of patients.

**Methods:**

This retrospective, single-center case series included 200 consecutive patients treated between 2014 and 2024. Adult patients with symptomatic or recurrent parastomal hernias were included; exclusion criteria were severe comorbidities precluding surgery or lack of follow-up data. Demographic and operative variables, perioperative outcomes, and recurrence rates were analyzed. Descriptive statistics were used (mean, SD, range); no hypothesis testing was applied.

**Results:**

The majority of patients had EHS Type III or IV hernias. The mean operative time was 171 min. In 10% of cases, a cost-effective “Baldachin modification” using polypropylene mesh was employed. Stoma relocation was required in 87% of Type IV cases. Postoperative complications occurred in 12.5%, primarily wound infections. The recurrence rate was 5.5%, and quality of life significantly improved (VAS score increased from 3.15 to 9.15). No mortality was observed.

**Conclusion:**

HyPER proved to be a safe and effective technique for treating parastomal hernias, especially in complex and recurrent cases. The approach allowed for thorough anatomical correction and yielded low recurrence rates with acceptable morbidity. The Baldachin modification may offer a viable low-cost alternative in resource-limited settings. Further multicenter studies are warranted to validate these findings and establish standardized protocols.

**Supplementary Information:**

The online version contains supplementary material available at 10.1007/s00464-025-12318-8.

Parastomal hernia (PH) is one of the most common and challenging complications following the creation of a stoma, with incidence rates up to 90% depending on the type of stoma and patient characteristics. Statistics indicate that up to 50% of parastomal hernias develop within the first two years after the initial surgery [1]. This percentage increases with the extension of patient survival, which is closely linked to the advancement of new oncological treatment techniques and improved overall life expectancy [1]. It significantly impacts patients’ quality of life, causing pain, discomfort, and difficulty with ostomy appliance management [2, 3]. Surgical repair remains the primary treatment option, with a variety of techniques available, including fascial repair, stoma relocation, and mesh-based methods like the Sugarbaker (both retrorectus and intraperitoneal) and keyhole techniques. Additionally, the literature includes reports presenting the above-mentioned techniques performed using laparoscopy or robotic surgery (laparoscopic Sugarbaker, Pauli method, etc.) [4–8].

The significance of parastomal hernia repair cannot be overstated, as the condition poses a growing population-level challenge. In the United States, approximately 725,000–1 million individuals live with a stoma, while in Poland, this figure is estimated at 70,000 [9]. Annual stoma creation procedures in Poland range from 7000 to 13,000. Notably, up to 50% of these patients will develop a parastomal hernia within the first two years post-surgery [10]. With advances in oncology extending patient survival, the burden of parastomal hernias continues to grow, necessitating effective and sustainable solutions.

The ongoing difficulties in achieving durable outcomes in parastomal hernia repair have prompted the search for innovative surgical solutions. The Hybrid Parastomal Endoscopic Repair (HyPER) technique combines the precision of laparoscopic surgery with the accessibility of open surgery to address the unique challenges of PH repair. The method developed by Szczepkowski et al. demonstrated promising preliminary results and appeared to be a significant tool in the context of parastomal hernia repair [11, 12]. Given that the technique has been performed at Bielański Hospital for over a decade, we aimed to summarize its effectiveness in a retrospective cohort study conducted on a large patient population.

This study aims to analyze the outcomes of 200 patients who underwent the HyPER procedure, focusing on recurrence rates, complication profiles, and patient satisfaction, while comparing the findings with existing literature. By doing so, it seeks to contribute to the growing body of evidence supporting optimized surgical strategies for managing parastomal hernias. This case series has been reported in line with the PROCESS 2025 Guideline [13].

## Materials and methods

This retrospective study was conducted at a high-volume tertiary referral center, primarily specializing in colorectal surgery and serving as a national reference unit for parastomal hernia management. A total of 200 patients with symptomatic parastomal hernias were included. All patients underwent the Hybrid Parastomal Endoscopic Repair (HyPER) technique, with surgical procedures performed by a team of experienced surgeons following a standardized protocol.

Inclusion criteria comprised adult patients presenting with symptomatic parastomal hernias, classified according to the European Hernia Society (EHS) system. Patients with disseminated malignancies, a short life expectancy, or contraindications to laparoscopy were excluded from the study. All procedures were conducted electively, with preoperative planning based on thorough clinical and imaging evaluations. Each patient underwent a CT scan before surgery, which was used to preoperatively classify the hernia into the appropriate EHS type.

**Fig. 1:**
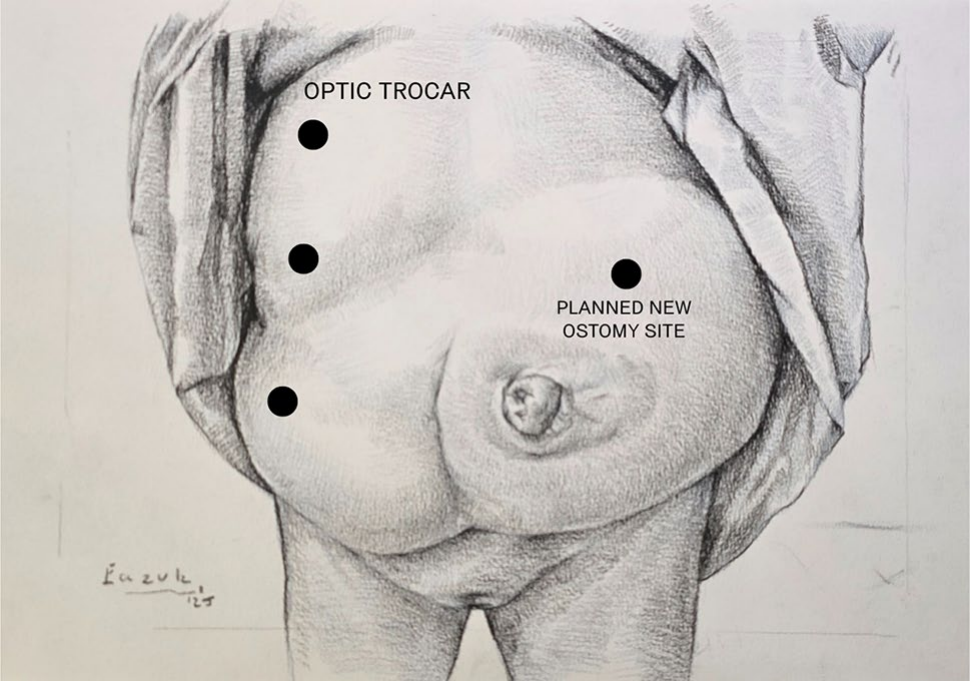
Preoperative planning and trocar placement

**Fig. 2:**
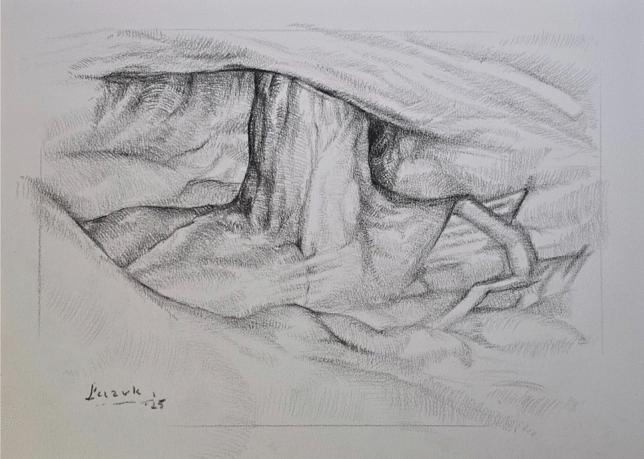
Laparoscopic stage—adhesiolysis

**Fig. 3:**
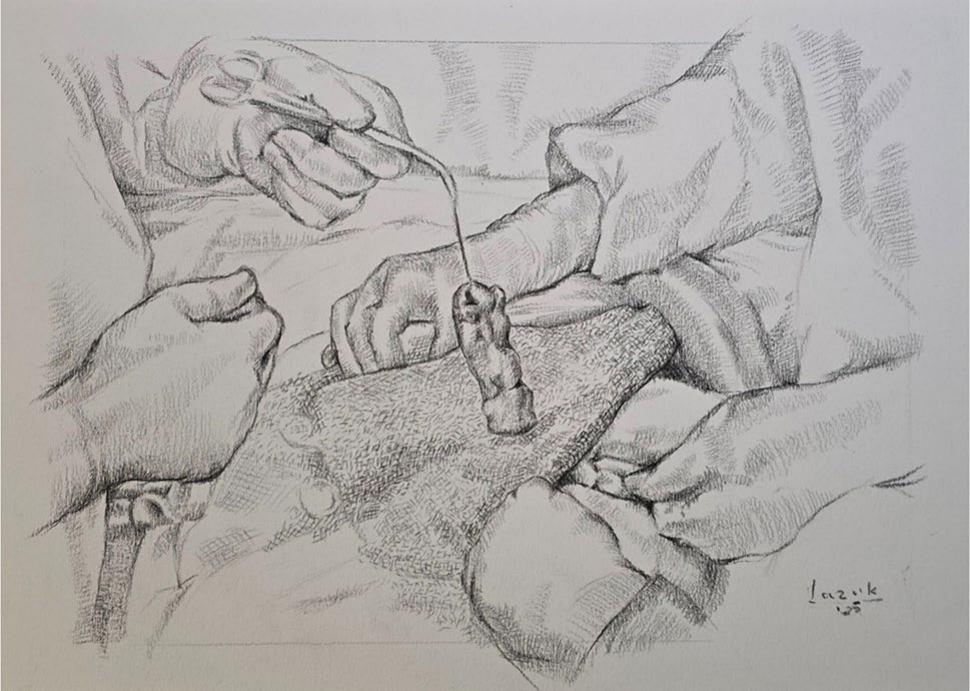
Open stage—hernia sac dissection and defect closure

**Fig. 4:**
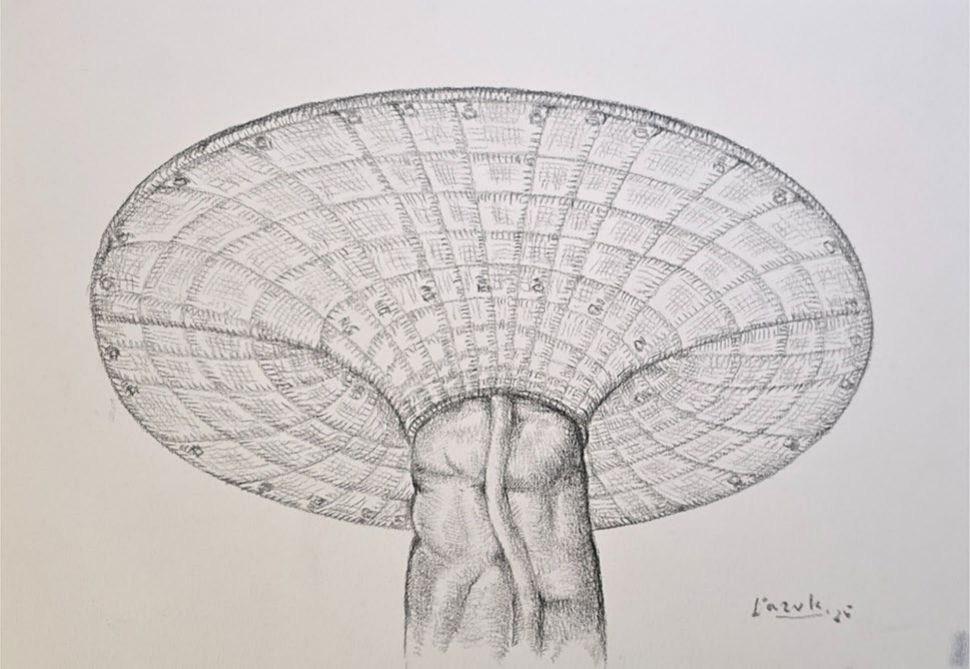
Mesh reinforcement and stoma exteriorization

Trocar placement is planned on the side opposite to the hernia, depending on stoma location. If stoma relocation is needed, a new site is selected preoperatively in consultation with the patient, considering body habitus and daily function. Site marking is performed in supine, standing, and seated positions to ensure optimal appliance fit (Fig. 1). The surgical technique was standardized across the cohort and followed a consistent four-step sequence. First, laparoscopic adhesiolysis was performed to mobilize the bowel and prepare the hernia site (Fig. 2). The open stage begins with a skin incision below the stoma, followed by sac dissection and removal. In EHS Type III and IV hernias, stoma loop mobilization is typically required, allowing the mesh to be threaded over the loop and positioned intraperitoneally. The fascial defect is then narrowed, and excess subcutaneous tissue resected (Fig. 3). Third, mesh reinforcement was achieved with laparoscopic intraperitoneal placement and fixation of a dedicated implant (Fig. 4). Finally, when indicated, the bowel was shortened and the stoma relocated or revised to improve tension and functional alignment. A detailed description of the method was previously published in BJS Open and Video surgery Journal [11, 12]. Method was also used to validate the EHS Parastomal Hernia Classification [14].

Polyvinylidene fluoride (PVDF) composite mesh (DynaMesh-IPST®, FEG Textiltechnik mbH, Aachen, Germany) was utilized for nearly all cases to ensure optimal reinforcement and reduced recurrence risk. Mesh fixation was performed using a permanent laparoscopic fixation system (CapSure®, BD, New Jersey, USA) in a double crown technique to provide stable and uniform mesh integration. In 20 cases, macroporous polypropylene mesh lacking an anti-adhesive layer was used (Optomesh® Stoma, TZMO, Łódź, Poland). In these cases, the omentum served as a separating layer between the implant and intestinal loops (Baldachin modification). The remaining surgical stages were identical to those performed with the PDVF composite implant.

All patients received perioperative care that included antibiotic prophylaxis and measures to mitigate thromboembolic risks. Postoperative follow-up ranged from 3 to 181 months, with a mean follow-up duration of 61 months. Patients were assessed for recurrence rates, complications, and quality of life using a Visual Analog Scale (VAS). Follow-up was consistently conducted in the hospital-based outpatient clinic and included a complete clinical examination by a surgeon. When clinically indicated, this assessment was supplemented by imaging studies, such as computed tomography (CT) or ultrasound (US), to ensure accurate detection of recurrence or complications.

The study was approved by the Bioethics Committee of the Regional Medical Chamber in Gdańsk (KB – 3/2024). Informed consent was waived due to the retrospective nature of the study. The study has been registered on the ClinicalTrials.gov platform under the identifier NCT07064694.

### Statistical analysis

This was a retrospective, single-arm study without a control group. Descriptive statistics were used to summarize all variables. Continuous data are presented as means with standard deviations or medians with ranges. Categorical variables are reported as counts and percentages. No hypothesis testing or inferential statistical analysis was performed. Given the retrospective nature of this case series, no a Priori statistical analysis protocol was created.

## Results

The study cohort consisted of 200 patients who underwent parastomal hernia repair using the HyPER technique between 2014 and 2024. The mean age at the time of surgery was 64.4 years (range: 38–86 years), with the majority of patients classified as ASA II (46.5%) or ASA I (26%), reflecting a population with moderate comorbidities suitable for elective surgical repair. The mean body mass index (BMI) was 28.04 kg/m^2^ (range: 19.1–40.3 kg/m^2^), indicating that most patients were overweight, which is typical for this surgical indication. The distribution of hernia types, classified according to the European Hernia Society (EHS), revealed a predominance of more complex presentations. Type III hernias accounted for the majority (56.5%), followed by Type IV (35%), while Type I and II hernias were less common (5.5% and 3%, respectively). This distribution underscores the referral nature of our center, where advanced or recurrent cases are frequently managed. Patient demographics, hernia characteristics, and clinical outcomes were collected and analyzed (Tables 1 and 2).

**Table 1:** Demographic data and patients characteristics

	Characteristics
Mean age (years) (range)	64.4 (28–88)
Sex	
Male	119 (59.5%)
Female	81 (40.5%)
Mean BMI (kg/m^2^) (range)	28.04 (19.53–39.06)
ASA classification:	
I	52 (26%)
II	97 (46.5%)
III	44 (22%)
IV	7 (3.5%)
Indications for stoma creation	Colorectal cancer – 131
	Inflammatory bowel disease – 10
	Other factors (fistulas, constipation, decubital ulcer, stool incontinence, prostate cancer) – 19
Indications for parastomal hernia repair	Difficulty with ostomy appliances – 38
	Parastomal hernia size – 87
	Poor cosmetic effect – 49
	Pain/discomfort – 141
	Episodes of intestinal obstruction – 32
Parastomal hernia orifice dimeter (cm) (range; SD)	7.61 (range: 2.7–15; SD ± 1.87)
Parastomal hernia sac diameter (cm) (range; SD)	17.8 cm (range 6–37; SD ± 5.8)

*BMI* body mass index, *ASA* American Society of Anesthesiologists, *SD* standard deviation

**Table 2:** Hernia types and outcomes

Type of hernia	Type I (*n* = 11)	Type II (*n* = 6)	Type III (*n* = 113)	Type IV (*n* = 70)
Soft tissue reconstruction	7 of 11 (60%)	5 of 6 (83.3%)	75 of 113 (66.4%)	56 of 70 (80%)
Stoma relocation	7 of 11 (60%)	5 of 6 (83.3%)	93 of 113 (82.3%)	61 of 70 (87%)
Stoma reduction	11 of 11 (100%)	6 of 6 (100%)	110 of 113 (97.3%)	67 of 70 (96%)
Average time of surgery (min) (range)	175.2 (90–295)	157 (100–190)	168.61 (90–345)	185.68 (105–300)
Postoperative complications (requiring rehospitalization)	–	Bleeding – 1 (16.7%)	SSI – 7 (6.19%) Intraoperative Bowel injury – 2 (1.8%) Hematomas – 3 (2.65%) Stoma necrosis – 1 (0.88%) Intestinal obstruction – 1 (0.88%)	SSI – 7 (10%) Hematomas – 3 (4.3%)
Recurrences (overall)	–	–	5 (4.42%)	6 (8.6%)
Recurrences (after 2-years follow-up) – total patients—141			4 (range: 2–4 years)	5 (range: 2 – 6 years)
Two-stage surgery	–	–	–	6 of 70 (8.6%)
Mean patient satisfaction before surgery (VAS scale)	3.23	2.8	3.38	3.18
Mean patient satisfaction after surgery (VAS scale)	8.82	9.2	9.39	9.19

*SSI* surgical site occurrence, *VAS* visual analog scale

Surgical complexity corresponded closely with hernia classification. The mean operative time was 171 min (range: 90–345 min). Patients with Type III and IV hernias experienced significantly longer operative durations, largely due to additional procedures such as stoma relocation or soft tissue reconstruction. For example, 87% of Type IV cases required relocation of the stoma due to defect position, redundancy of bowel loops, or skin complications. In 80% of these patients, simultaneous panniculectomy or abdominoplasty was performed to eliminate redundant tissue, which hindered proper stoma appliance fitting and could predispose to recurrence. Stoma loop shortening was performed in 97% of patients to eliminate excess length, which is a known contributor to hernia recurrence and poor pouch adhesion. This step was deemed essential in nearly all Type III and IV cases. Overall, the HyPER technique allowed for a comprehensive anatomical correction including fascial defect closure and mesh reinforcement.

Mesh selection was individualized. PVDF composite mesh was used in most patients, with 123 receiving a 15 × 15 cm mesh, 54 receiving 25 × 25 cm, and 3 receiving 30 × 30 cm variants. In 20 patients (10%), the Baldachin modification was performed with a 19.5 × 19.5 cm macroporous polypropylene mesh serving as a cost-effective option. In these cases, the greater omentum was interposed to prevent direct contact between the mesh and bowel (Table 3).

**Table 3:** Hernia types and meshes used

Category	Description	*n*
Mesh used	PDVF composite 15 × 15 cm	123 (61.5%)
	PDVF composite 25 × 25 cm	54 (27%)
	PDVF composite 30 × 30 cm	3 (1.5%)
	Macroporous polypropylene 19.5 × 19.5 cm	20 (10%)
Type of stoma present	End colostomy	176 (88%)
	Urostomy	9 (4.5%)
	Loop colostomy	11 (5.5%)
	End ileostomy	4 (2%)
Hernia type	Primary parastomal hernia	185 (92.5%)
	Recurrent parastomal hernia	15 (7.5%)

*n* = number of patients

Regarding stoma type, 176 patients (88%) had an end colostomy, while 11 (5.5%) had loop colostomies, 9 (4.5%) had urostomies, and 4 (2%) had end ileostomies. The majority of repairs (92.5%) were for primary hernias, while 15 patients (7.5%) presented with recurrence after previous repair.

Postoperative complications occurred in 12.5% of the cohort (*n* = 25), and were more frequent among patients with complex hernias (Types III and IV). The most common complication was surgical site infection (7%), followed by seroma formation and minor wound dehiscence. Major complications, such as stoma necrosis and intraoperative bowel injury, were rare and occurred exclusively in patients with Type IV hernias. There were no cases of mesh-related enterocutaneous fistula or early mesh explantation.

The recurrence rate in the overall cohort was 5.5% (11 patients), observed only in Type III (5/113; 4.4%) and Type IV (6/70; 8.6%) hernias. No recurrences were reported in Type I or II cases. Notably, among patients who received the Baldachin modification, no early recurrence was observed during the first 12 months of follow-up, and the complication rate was 15%, comparable to the 14.8% in the PVDF composite mesh group.

Two-stage repair was performed in three patients with EHS Type IV hernias, representing 4.3% of all Type IV cases. This approach was used in patients presenting with both a massive midline incisional hernia and a large parastomal hernia, a two-stage surgical approach was adopted. When a fascial bridge was present between the defects, initial repair focused on the midline hernia (Rives-Stoppa technique), followed by a delayed parastomal hernia repair approximately six months later. The initial repair provided a structural foundation for the subsequent procedure and allowed us to avoid implanting an excessively large intraperitoneal mesh, which is rarely an optimal solution.

Quality of life (QoL) was assessed using a visual analog scale (VAS) before and after surgery. Preoperative scores averaged 3.15 (SD ± 1.1), indicating moderate-to-severe impairment. Postoperative scores averaged 9.15 (SD ± 0.6), reflecting a significant subjective improvement in both physical comfort and psychosocial well-being. Follow-up was robust: 141 patients (70.5%) completed at least 2 years of follow-up. Durability of repair was assessed through clinical examination, standardized imaging (including CT scans with Valsalva maneuver), and patient-reported outcomes.

## Discussion

The problem of parastomal hernias remains far from being resolved. The literature reveals a variety of, often conflicting, approaches to the issue [1, 6]. Preventive measures, such as the implantation of mesh during the initial stoma creation, initially showed promising results [2]. However, recent meta-analyses have questioned these outcomes [15]. There is also a growing trend towards minimal interventions, including a return to the onlay technique or simple suturing, due to the lack of consensus on the optimal treatment strategy for parastomal hernias [16]. Proponents argue that given the high recurrence rates seen with other methods (e.g., Sugarbaker, modified keyhole), a less invasive and less burdensome approach might be more appropriate for patients.

Robotic surgery has also entered the field of parastomal hernia repair, with reports on robotic Sugarbaker or robotic Pauli procedures [4, 17, 18]. While short-term outcomes are promising, the lack of long-term results, which are critical for parastomal hernias, remains a significant limitation. Another issue frequently observed in studies is the lack of a standardized classification system, making it unclear whether the repairs involved massive defects often requiring staged reconstruction, such as Type IV, or smaller parastomal hernias like Type I. Although the EHS classification for parastomal hernias provides a valuable framework for standardizing defect description and surgical planning, it remains underutilized in routine clinical reporting. In our study, we applied the EHS system prospectively to all 200 cases and analyzed outcomes accordingly, offering new insight into recurrence risk and complexity stratification.

This study presents the largest known single-center cohort of parastomal hernia repairs using a standardized surgical protocol, contributing a uniquely robust dataset to the existing body of literature. This fact should not be overlooked. Existing literature has thus far been limited to small patient cohorts and has often failed to adequately characterize the hernias being treated. In particular, the European Hernia Society (EHS) classification—which remains the only validated system for abdominal wall hernias—has rarely been applied, making it difficult to compare outcomes across studies [14]. The only publication approaching the volume and follow-up duration of our series is that of Uhlback-Makarainen et al. However, it must be emphasized that their study was retrospective, conducted nationwide across five referral centers, and primarily highlights the limitations and high recurrence rates associated with previously used surgical techniques [19]. Additionally, it is worth noting the studies by De Robles et al. and Xiaojian Fu et al., which, although smaller in scale, report relatively large series of parastomal hernia repairs (76 and 81 cases, respectively) [20, 21].

One of the key strengths of the HyPER technique lies in its hybrid nature, combining the precision of laparoscopic surgery with the accessibility and flexibility of open surgery.

This dual approach allows for thorough removal of the hernia sac—often an overlooked step in other surgical techniques—and comprehensive soft tissue reconstruction. The latter includes the excision of excess, damaged skin through panniculectomy and creating a new skin site for stoma relocation. Critical elements contributing to the success of the HyPER technique include narrowing the hernia defect, reinforcing the area with a large implant, and shortening the redundant afferent bowel loop to the required length. This comprehensive strategy contrasts with several widely used techniques—such as the traditional Sugarbaker, keyhole, or sandwich approaches—which often prioritize minimal manipulation of the stoma and bowel to reduce operative risk [7, 8, 22]. While such conservative methods may limit immediate morbidity, they may fail to address the underlying anatomical drivers of hernia formation, such as fascial widening, stomal prolapse, or redundancy of the bowel loop. In the HyPER approach, targeted loop shortening eliminates excess tension and reduces the risk of stomal telescoping or lateral bulging, while defect narrowing and sac excision restore the integrity of the abdominal wall. This contrasts with the “stoma-sparing” philosophy that treats the ostomy site as sacrosanct and leaves potentially pathogenic elements unaddressed [23]. We believe that the presence of a colorectal surgeon is essential in parastomal hernia repairs, particularly due to their expertise and lack of hesitation in modifying or relocating the stoma when necessary—a factor increasingly supported by recent literature emphasizing the importance of multidisciplinary involvement in complex abdominal wall reconstruction [24].

### Reconsidering the 5-cm threshold

The 5-cm cut-off used by the EHS to differentiate between small and large parastomal hernias may not adequately reflect patient-specific factors such as body size and composition. For larger individuals, a 5-cm defect might be relatively minor, whereas in smaller patients, it could constitute a significant proportion of the abdominal wall. A more individualized approach, such as calculating the defect size relative to the total anterior abdominal wall surface (using CT imaging), could better capture the clinical significance of the hernia size.

### Type IV hernia issue

In Type IV hernias, the size of the defect is not the only factor influencing surgical strategy. The presence or absence of a fascial bridge connecting the parastomal hernia to other hernias plays a critical role. A single large hernia without a fascial bridge often necessitates a one-stage repair, while the presence of a fascial bridge (minimum 3 cm) enables a two-stage approach. Our preferred method involves addressing the midline hernia first, followed by parastomal hernia repair after 3–6 months. This approach is supported by biomechanical evidence favoring retromuscular repairs with a minimum 3-cm fascial separation [25–27].

### Role of imaging

Although CT imaging is instrumental in evaluating parastomal hernias, it often underestimates the true defect size by as much as 20%. This discrepancy highlights the need for surgeons to anticipate larger-than-expected defects during surgical planning. EHS parastomal hernia classification relies on clinical findings supported by CT data, but the imaging limitations emphasize the importance of intraoperative reassessment for accurate repair strategies. Our observations are consistent with previously published data, confirming that CT imaging performed without a Valsalva maneuver may be insufficient for accurate assessment of parastomal hernias [29].

### Cost considerations and the Baldachin modification

An important aspect often overlooked in discussions surrounding mesh selection is the availability of cost-effective alternatives to advanced implants. In 2023, we introduced a novel technique termed the Baldachin modification to address the challenge of limited access to high-cost meshes. This approach utilizes a macroporous polypropylene mesh without an anti-adhesive barrier, which is fashioned into a tunnel-like configuration that mimics the functional architecture of the PVDF composite meshes. To reduce the risk of adhesions between the mesh and bowel loops, a flap of the greater omentum was interposed whenever feasible, serving as a biological barrier. This omental flap was positioned to underlay the mesh, effectively separating it from the underlying intestinal loops and minimizing the risk of direct contact and adhesion formation. This technique was applied in 20 patients within our cohort. In this subgroup, the 30-day postoperative complication rate was 15%, including one seroma and two minor wound infections. No mesh-related complications or early recurrences were observed. These outcomes were comparable to those in the PVDF mesh group (180 patients), where the complication rate was 14.8% and no early recurrences were noted within the first 3 months. However, the mean follow-up in the Baldachin subgroup was shorter (12 months vs. 64 months), and long-term data are not yet available. The technique also has clear limitations. In patients with a history of extensive intra-abdominal surgery or partial omentectomy—which may have been performed during prior oncological procedures such as cytoreductive surgery or colectomy—the greater omentum is often absent or immobile, making it impossible to establish an effective interpositional barrier. In such scenarios, the Baldachin modification is contraindicated, and alternative techniques must be considered. Importantly, the cost of the polypropylene mesh used in the Baldachin modification is approximately ten times lower than that of the PVDF composite mesh, making it a promising option in resource-limited healthcare settings. Further prospective studies are needed to validate this technique in larger populations and over longer follow-up periods, particularly regarding adhesion-related complications and recurrence.

### Follow-up and mortality considerations

The mean follow-up duration in this study was 61 months, with approximately 60% of patients being observed for more than 5 years and over 85% assessed during the critical initial two-year postoperative period. This extended longitudinal observation enhances the reliability of our recurrence and complication data. Nonetheless, a proportion of patients died during the follow-up period due to unrelated causes, reflecting the advanced age and comorbid status of the study population. While exact mortality numbers are available in the source data, it is important to note that none of the recorded deaths were attributable to the surgical procedure itself or its complications.

### Rethinking priorities

One of the key challenges in the treatment of parastomal hernias—and in the reporting of clinical outcomes—lies in the almost exclusive focus on recurrence rates and local complications such as wound infections or seromas. While these aspects are certainly important, it must be emphasized that a crucial component of successful treatment is the patient’s postoperative quality of life. For individuals living with a stoma, baseline quality of life is often already significantly impaired. Therefore, beyond eliminating the hernia itself, it is equally important to create conditions that enable patients to live with a stoma in a way that is as unobtrusive as possible. This includes the ability to properly apply and maintain ostomy appliances and baseplates with minimal difficulty or skin irritation.

For this reason, the authors take a critical view of approaches that limit themselves solely to minimally invasive techniques in parastomal hernia repair. It must be noted that in the vast majority of cases—especially in EHS Type III and IV hernias—we are dealing with an excess of deformed and often damaged skin and subcutaneous tissue. Leaving this issue unaddressed—without performing soft tissue reconstruction, stoma shortening when necessary, or stoma relocation—can result in a situation where the patient’s quality of life remains extremely poor, despite the hernia being technically resolved.

Such patients continue to struggle with issues such as ostomy pouch detachment in public settings, chronic skin irritation, ulcerations, and the need to improvise solutions just to keep their stoma appliances in place. For this reason, the authors firmly believe that complete excision of the hernia sac and panniculectomy (resection of redundant skin and subcutaneous fat) are just as important as definitive closure of the hernia defect itself. These essential steps are simply not achievable through purely minimally invasive approaches such as laparoscopy or robotic surgery.

Therefore, in the vast majority of cases presented in this study, patients underwent soft tissue reconstruction in conjunction with simultaneous stoma relocation, always on the same side as the original stoma site, but in a better location than previously. The new stoma site was each time marked and discussed with the patient prior to surgery, taking into account anatomical changes resulting from the removal of the hernia sac and the excess skin and subcutaneous fold. Where necessary, the procedure also included shortening of the intestinal loop. Only such a comprehensive approach leads to full success in treating the complex condition that is a parastomal hernia.

### Limitations

This study has several limitations inherent to its retrospective, single-center design. The relatively large cohort is an advantage, but the absence of a control group precludes direct comparison with alternative surgical techniques. Selection bias is possible, as only patients eligible for the HyPER approach were included. The Baldachin modification, although promising, was performed in a limited subgroup and requires validation in larger, prospective cohorts. Finally, the absence of randomization and reliance on descriptive statistics limit the strength of causal inferences. The generalizability of these findings is also subject to contextual factors. Our institution is a high-volume tertiary referral center specializing in colorectal surgery and serving as the national reference unit for parastomal hernia management. This explains the predominance of advanced hernia types (EHS Type III and IV) in the cohort, emphasizing the technique’s utility in complex scenarios but limiting extrapolation to less severe cases. Moreover, while the surgical protocol was standardized, outcomes may vary depending on surgeon expertise and the availability of specific implants, including PVDF composite meshes or cost-effective alternatives such as the Baldachin modification.

With a recurrence rate of 5.5% and significant postoperative improvement in patient-reported quality of life, the results support HyPER as a viable and durable approach to PH repair. Key technical elements—including thorough hernia sac excision, mesh reinforcement, stoma loop shortening, and soft tissue reconstruction—appear to play a critical role in achieving favorable outcomes.

Further multicenter studies and randomized controlled trials are needed to confirm the reproducibility of these results and to define the optimal role of the HyPER technique within the broader context of parastomal hernia management. Consideration of cost-effectiveness, especially in relation to mesh choice and the potential scalability of modifications like the Baldachin method, will also be essential for broader adoption.

## Conclusions

The HyPER technique proved to be a safe, effective, and reproducible surgical approach for the treatment of parastomal hernias, particularly in complex and recurrent cases. The low recurrence rate, acceptable complication profile, and significant improvement in quality of life observed in this large cohort supported its continued use and further investigation. Moreover, the proposed Baldachin modification emerged as a promising cost-effective alternative to expensive specialized meshes, warranting additional long-term evaluation. Future studies should aim to validate these findings in broader clinical settings and define standardized protocols to enhance the generalizability of outcomes.

## Supplementary Information

Below is the link to the electronic supplementary material.

Supplementary file1 (DOCX 32 kb)

## Data Availability

All data will be presented on reasonable request.
