# Supplementary material for: Hybrid parastomal endoscopic repair (HyPER): a retrospective case series of 200 patients treated over ten years at a single center
Source: Surg Endosc. 2025 Nov 25;40(2):1479–87. doi: 10.1007/s00464-025-12318-8 (PMC12881038; doi:10.1007/s00464-025-12318-8)
Supplement: Supplementary file 1 — Supplementary file1 (DOCX 32 kb) [file 464_2025_12318_MOESM1_ESM.docx]

| **PROCESS 2025 Guideline Checklist** | | |  |
| --- | --- | --- | --- |
| **Topic** | **Item** | **Item description** | **Page Number** |
| **Title** | 1 | - The phrase ‘case series’ is included - The focus of the research study is mentioned (e.g. patient population, setting, diagnosis, intervention, outcome etc.) | 1 |
| **Key Words** | 2 | - Include three to six keywords that identify what is covered in the case series (e.g. patient population, setting, diagnosis, intervention, outcome etc.) - Include ‘case series’ as one of the keywords - Include the surgical subspecialty the case series pertains to as a keyword | 2 |
| **Abstract** | 3a | Introduction – briefly describe:   - Background - Scientific rationale for this study - Overarching theme of the case series - Aims and objectives | 2 |
|  | 3b | Methods – briefly describe:   - Sample size - Timeframe of research - Characteristics of study design (e.g. prospective/retrospective, single-/multi-centre, informal/formal, consecutive/non-consecutive, exposure-/outcome-based sampling, clinical/population-based etc.) - Inclusion and exclusion criteria | 2 |
|  | 3c | Results – briefly describe:   - Outcomes of the intervention/management strategy - Analysis - narrative or statistical (report any statistical testing, although mostly inappropriate in case series studies) | 2 |
|  | 3d | Conclusion – briefly describe:   - Key findings and take-home messages - Impact on future clinical practice - Direction of future research | 2 |
|  | 3e | Present a structured abstract   - Informal case series – introduction, case presentations (brief description of each case) and discussion/conclusion - Formal case series – introduction, methods, results and discussion/conclusion | 2 |
| **Highlights** | 4 | - Convey the key findings of the research study in 3 to 5 bullet points | 3 |
| **Artificial Intelligence (AI)**  (some journals may prefer this in the methods and/or acknowledgments section and it should also be declared in the cover letter) | 5 | Declaration of whether any AI was used in the research and manuscript development   - If no, proceed to item 6. - If yes, proceed to item 5a | 12 |
|  | 5a | **Purpose and Scope of AI Use**   - Precisely state why AI was employed (e.g. development of research questions, language drafting, statistical analysis/summarisation, image annotation, etc). - Was generative AI utilised and if so, how? - Clarify the stage(s) of the reporting workflow affected (planning, writing, revisions, figure creation).   Confirmation that the author(s) take responsibility for the integrity of the content affected/generated. | n/a |
|  | 5b | **AI Tool(s) and Configuration**   - Name each system (vendor, model, major version/date). - State the date it was used - Specify relevant parameters (e.g. prompt length, plug-ins, fine-tuning, temperature). - Declare whether the tool operated locally on-premises, or via a cloud API and any integrations with other systems. | n/a |
|  | 5c | **Data Inputs and Safeguards**   - Describe categories of data provided to the AI (patient text, de-identified images, literature abstracts). - Confirm that all inputs were de-identified and compliant with GDPR/HIPAA. - Note any institutional approvals or data-sharing agreements obtained. | n/a |
|  | 5d | **Human Oversight and Verification**   - Identify the supervising author(s) who reviewed every AI output. - Detail the process for fact-checking, clinical accuracy checks - State whether any AI-generated text/figures were edited or discarded. - Acknowledge the limitations of AI and its use | n/a |
|  | 5e | **Bias, Ethics and Regulatory Compliance**   - Outline steps taken to detect and mitigate algorithmic bias (e.g. cross-checking against under-represented populations). - Affirm adherence to relevant ethical frameworks.   Disclose any conflicts of interest or financial ties to AI vendors. | n/a |
|  | 5f | **Reproducibility and Transparency**   - Provide the exact prompts or code snippets (as supplementary material if lengthy). - Supply version-controlled logs or model cards where possible. - If applicable, state repository, hyperlink or digital object identifier (DOI) where AI-generated artefacts can be accessed, enabling attempts at independent replication of the query/input. | n/a |
| **Introduction** | 6 | **Introduction** comprehensively describe:   - Relevant background and scientific rationale for case series with reference to key scientific literature - Overarching theme (e.g. common patient population, setting, diagnosis, intervention, outcome etc.) - Aims and objectives - At the end of the introduction, refer to the PROCESS 2025 publication by stating: ‘This case series has been reported in line with the PROCESS guidelines [include citation]’ | 4 |
| **Methods** | 7a | **Participants** comprehensively describe:   - Relevant participant characteristics (e.g. demographics, comorbidities, ASA score, severity of surgery, urgency of surgery, smoking status, tumour staging etc.) and if relevant, exposure(s) of the participants (e.g. COVID-19) - Subsequent inclusion and exclusion criteria with clear definitions - Approach to selecting patients (e.g. consecutive/non-consecutive, exposure-/outcome-based, formal/informal etc.)   Methods used to ensure de-identification of patient information | 5 and 6 |
|  | 7b | **Recruitment** comprehensively describe:   - Sources of recruitment (e.g. physician referral, electronic health record etc.)   Any monetary incentivisation of patients for recruitment and retention should be declared; clarify the nature of any incentives provided | 6 |
|  | 7c | **Pre-intervention patient optimisation:**   - Lifestyle (e.g. weight loss, nutritional support, exercise, smoking cessation etc.) - Medication review (e.g. anticoagulation, oral hypoglycemics, insulin, oral contraceptive pill etc.) - Pre-surgical stabilisation/preparation (e.g. treating hypothermia/-volemia/-tension, ICU care, nil by mouth, bowel preparation etc.)   Other (e.g. psychological support, pre-operative education/counselling etc.) | 5 and 6 |
|  | 7d | **Interventions** comprehensively describe:   - Type of intervention (e.g. pharmacological, surgical, physiotherapy, psychological etc.) - Aim of intervention (preventative/therapeutic) - Concurrent treatments (e.g. antibiotics, analgesia, antiemetics, venous thromboembolism prophylaxis etc.) | 5 and 6 |
|  | 7e | **Intervention specifics** comprehensively describe:   - Rationale for the treatment offered - Techniques involved in the administration of the intervention - Time to intervention - For pharmacological therapies, include details such as formulation, dosage, strength, route and duration - For surgical intervention, include details on anaesthesia, patient positioning, preparation used, equipment needed, devices, sutures, surgical stage etc. - Degree of novelty of surgical technique/device (e.g. 'first in human' or 'first in this context') - Manufacturer and model of any medical devices used | 5 and 6 |
|  | 7f | **Operator details** comprehensively describe:   - Relevant training, specialisation and operator’s experience (e.g. average number of the relevant procedures performed annually, independent, needs direct/indirect supervision etc.) - Learning curve for technique - Requirement for additional training - Collaboration with other specialities (e.g. hybrid cardiac surgery) | 5 |
|  | 7g | **Quality control** comprehensively describe:   - Measures taken to reduce inter- or intra-operator/operation variation, ensure quality and maintain consistency between cases (e.g. independent observers, lymph node counts, standard surgical technique etc.) - Any specific disparities between cases | 5 |
|  | 7h | **Post-operative care and follow-up** comprehensively describe:   - Post-operative care (e.g. patient education, post-operative medications, early mobilisation, targeted physiotherapy, early enteral nutrition, early removal of catheters/drains, psychological therapy etc.) - Follow-up timeframes (e.g. first follow-up post-discharge, follow-up duration at the time of submission etc.) and frequency - Follow-up setting (e.g. home via phone/video consultation, primary care, secondary care etc.) - Follow-up method (e.g. history, clinical examination, blood tests, imaging etc.) - Follow-up personnel (e.g. operating surgeon) - Any specific long-term surveillance requirements (e.g. imaging surveillance of endovascular aneurysm repair, clinical/ultrasound examination of regional lymph nodes for skin cancer etc.) - State if any participants were lost to follow-up and why | 5 and 6 |
|  | 7i | **Analysis**   - Narrative or statistical (report any statistical testing, although mostly inappropriate in case series studies) | 6 |
| **Results** | 8a | **Participants** comprehensively describe:   - Number of patients involved - Patient characteristics (e.g. demographics, comorbidities, ASA score, severity of surgery, urgency of surgery, smoking status, tumour staging etc.) and if relevant, exposure(s) of the participants (e.g. COVID-19) - Include table showing baseline patient characteristics | 6 |
|  | 8b | **Deviation from the initial management plan** comprehensively describe:   - Any changes to the planned intervention with rationale - If appropriate, include a suitable schematic diagram | n/a |
|  | 8c | **Outcomes and follow-up** comprehensively describe:   - Expected versus attained clinician assessed outcome, providing reference to scientific literature used to inform expected outcomes (e.g. core outcome set) - If appropriate, include patient-reported outcomes (e.g. quality-of-life) - Use of validated outcome measures - Time of outcome occurrence - Percentage of patients lost to follow-up with rationale | 6 and 7 |
|  | 8d | **Intervention adherence and compliance** comprehensively describe:   - Assessment of patient’s adherence and tolerability of intervention and post-operative instructions (e.g. avoiding heavy lifting/strenuous activity, tolerance of chemotherapy/pharmacological agents etc.) - Impact on long-term applicability of intervention in clinical practice | 6 and 7 |
|  | 8e | **Complications and adverse events** comprehensively describe:   - Precautionary measures taken to prevent complications (e.g. antibiotic/venous thromboembolism prophylaxis) - Complications and adverse events (e.g. blood loss, wound infection, deep vein thrombosis, pulmonary embolism etc.), categorised in accordance with the Clavien-Dindo classification - Timing of adverse events - Mitigation for adverse events (e.g. blood transfusion, wound care, re-exploration/revision surgery etc.) - If relevant, whether complications or adverse events were discussed locally (e.g. morbidity and mortality meetings) - If appropriate, whether complications or adverse events were reported to the relevant national agency or pharmaceutical company - Specify time to discharge following completion of intervention and whether this was within the expected timeframe or not (if not, why not) - Where applicable, specify the 30-day post-operative and long-term morbidity/mortality - State if there were no complications or adverse events | 6 and 7 |
| **Discussion** | 9a | **Key results**  comprehensively describe:   - Key results - Include table showing key results | 6 and 7  Table 1,2,3 |
|  | 9b | **Scientific context and implications** comprehensively describe:   - Relevant literature and if appropriate, similar published studies - Implications for clinical practice and guidelines (e.g. NICE) - Comparison to current gold standard of care - Relevant hypothesis generation | 7 |
|  | 9c | **Strengths**  comprehensively describe:   - Strengths of the study - Any multidisciplinary or cross-speciality relevance | 8-11 |
|  | 9d | **Weaknesses and limitations**  comprehensively describe:   - Weaknesses and limitations of the study, with potential impact on results and their interpretation - Deviations from protocol, with reasons - For novel techniques or devices, outline any contraindications/alternatives and potential risks/complications if applied to a larger population | 11 |
|  | 9e | **Directions for future research**  comprehensively describe:   - Impact on future research and clinical practice - Questions that have arisen as a result of the study - Alternative study design(s) best suited to address these questions | 11 and 12 |
|  | 9f | **Cost**  comprehensively describe:   - Economic implication(s) - Justify cost if intervention more expensive than current gold standard of care - Any cheaper alternatives | 9 |
| **Conclusions** | 10a | **Key conclusions**   - Outline the key conclusions from this study | 12 |
|  | 10b | **Rationale**   - Explain the rationale behind those conclusions | 12 |
|  | 10c | **Future work**  briefly describe:   - Any questions arisen from the study - Any differences in approach to patient diagnosis or management which authors might adopt in future similar studies | 12 |
| **Patient and/or Carer Perspective** | 11 | - Where appropriate, the patient(s)/carers(s) should be given the opportunity to share their perspective on the intervention(s) they received (e.g. sharing quotes from a consented, anonymised interview or questionnaire) | 6 |
| **Informed Consent** | 12 | - The authors must provide evidence of consent, where applicable, and if requested by the journal - State the method of consent at the end of the article (e.g. verbal or written) - If not provided by the patients, explain why (e.g. death of patient and consent provided by next of kin). If the patients or family members were untraceable then document the tracing efforts undertaken. | 6 |
| **Additional Information** | 13a | - State any conflicts of interest | 12 |
|  | 13b | - State any sources of funding (e.g. grant details) - Role of funder | 12 |
|  | 13c | Other relevant disclosures   - State any author contributions and acknowledgments - If appropriate, give details of institutional review board and ethical committee approval - Disclose whether the case has been presented at a conference or regional meeting | 12 |
| **Clinical Images and Videos** | 14 | - Where relevant and available, include clinical images to help demonstrate the cases pre-, peri- and post-intervention (e.g. radiological, histopathological, patient photographs, intraoperative images etc.) - Where relevant and available, include a link (e.g. Google Drive, YouTube etc.) to the narrated operative video to highlight specific techniques or operative findings - Ensure all media files are appropriately captioned and indicate points of interest to allow for easy interpretation | Figures 1-4 |
| **Referencing the Checklist** | 15 | - Include reference to the PROCESS 2023 publication by stating: 'This case series has been reported in line with the PROCESS Guideline' at the end of the methods section and include citation in the references section | 13 |
